# Impact of pooled procurement of medicines on patient adherence and economic burden: evidence from China

**DOI:** 10.7189/jogh.15.04229

**Published:** 2025-08-22

**Authors:** Boya Zhao, Jing Wu, Zhao Cheng, Xing Lin Feng

**Affiliations:** 1 School of Public Health, Peking University, Beijing, China; 2 School of Pharmaceutical Science and Technology, Tianjin University, Tianjin, China; 3 Department Centre for Social Science Survey and Data, Tianjin University, Tianjin, China

## Abstract

**Background:**

Pooled procurement is widely adopted to improve medicine affordability, yet its impact on patients’ medicine adherence and economic burden remains underexplored. This study addresses these gaps under China’s National Volume-Based Procurement (NVBP) policy.

**Methods:**

Using claims data from Tianjin, China (2017–2020), we conducted a retrospective cohort study of new-users of NVBP antihypertensive medicines before and after policy implementation. During one-year follow-up since medicine initiation, medicine adherence (measured by proportional days covered), medicine persistence (measured by discontinuation rate), and direct medical costs (broken down by components) were compared between cohorts. Linear regressions, Cox proportional hazard models and generalised linear models were used to analyse outcome differences, adjusting for patients’ demographics, medical history, and prior health care utilisation.

**Results:**

We identified 14 560 and 18 858 patients in pre- and post-policy cohorts, respectively (mean age = 57.1; 52.8% men). Compared to the pre-policy cohort, the post-policy cohort presented a slight increase in adherence to NVBP medicines (proportional days cover = 0.31 *vs*. 0.28; adjusted difference = 0.021; *P* < 0.0001), but no significant change in discontinuation rate. Hypertension-related costs decreased by 19.5% (1509.2 *vs*. 1804.8 Chinese Yuan; *P* < 0.0001) for the post-policy cohort, entirely attributed to saving in costs of NVBP medicines (480.6 *vs*. 772.6 Chinese Yuan; adjusted difference = −47.5%; *P* < 0.0001). No significant difference was observed in costs for other medicines and services. The cost saving was equally borne by patients and health plans.

**Conclusions:**

China’s NVBP modestly improved adherence and significantly reduced the economic burden for patients. To fully deliver patient-centred benefits of pooled procurement for chronic disease medicines beyond price cuts, it should be paired with supply- and demand-side auxiliary measures. China’s experience, including financial incentives, regulatory oversight, favourable reimbursement policies and public campaigns, may offer lessons for other settings. Longer-term studies in broader populations are needed to further research.

Access to safe, quality-assured, effective, and affordable medicines is essential for achieving universal health coverage. While, high medicine prices remain a major barrier to this goal globally [1]. To improve medicine affordability for both patients and health care systems, many countries/regions have adopted various pharmaceutical pricing policies, among which pooled procurement has received growing attention [1]. Pooled procurement consolidates the resources across multiple purchasing authorities to form a single purchasing entity, thereby enhancing purchasing power and procurement efficiency to reduce medicine prices [1,2]. It has been widely adopted at national and subnational levels in countries such as France, India, Brazil, Colombia, and China [3–8], and through international initiatives such as the Eastern Caribbean Drug Service and the Global Fund to Fight AIDS, Tuberculosis, and Malaria [9,10]. These experiences consistently demonstrate that pooled procurement effectively reduces medicine prices, leading to substantial savings in pharmaceutical expenditures [3–12].

Despite its wide application, evidence on patient-level effects of pooled procurement remains limited. In particular, it is unclear how physician’s prescribing behaviours or patients' medicine usage behaviours respond to this policy, and how such responses may influence adherence and costs. In the setting of other pharmaceutical pricing policies, studies have shown that lowering medicine prices can significantly improve medicine adherence [13–16], a key factor in managing chronic diseases. However, evidence from pooled procurement contexts is still lacking. To date, only one study in China has found a modest improvement in adherence, focusing solely on patients who underwent generic substitution for amlodipine, limiting its generalisability [17]. Moreover, previous research on medicine procurement has raised concerns about a phenomenon known as ‘reallocation of demand’, where patients’ savings from low-priced medicines may be offset by increased prescriptions of alternative medicines not covered by the policy [18]. In the health economics literature, this is also referred to as the ‘substitution effect’, reflecting providers’ behavioural responses to price changes [19]. Although some patient-level studies have found reductions in per-visit costs or household health care expenditure after pooled procurement, they did not break down the cost components to generate a deeper understanding, and relying on time series or cross-sectional designs limits their ability of causal inference [17,20–23].

Crucially, few studies have explored how specific policy design features of pooled procurement may shape these patient-level outcomes. In practice, pooled procurement is typically implemented alongside auxiliary measures [1], such as tendering or negotiation rules, provider incentives, or reimbursement adjustments, that vary across institutional and national contexts. Understanding how such policy designs may shape patient-level outcomes is essential to realising the full potential of pooled procurement as a patient-centred reform.

China initiated its national-level pooled procurement for medicines, known as National Volume-Based Procurement (NVBP), in late 2018. Since then, NVBP has become the country’s main pharmaceutical pricing policies for non-innovative medicines. This provides a value opportunity to addresses the knowledge gaps outlined above. This study aims to evaluate the impact of NVBP on patient adherence and economic burden, focusing on antihypertensive medicines, which not only represented the largest therapeutic category (30%) in the first NVBP round but also had all guideline-recommended pharmacological classes covered by the policy. Given the high prevalence of hypertension in China (27.5% in 2018) and its chronic treatment nature, antihypertensive medicines serve as a representative case for policy evaluation [24]. We adopted a cohort design and compared new users of antihypertensive NVBP medicines either before or after policy implementation, reducing the selection bias related to disease progression or treatment incentives. We also discuss how policy design within pooled procurement can mitigate substitution effects and enhance patient-centred outcomes.

## METHODS

This study was reported in accordance with the Strengthening the Reporting of Observational Studies in Epidemiology guidelines (Checklist S1 in the **Online Supplementary Document**).

### Setting

In early 2019, the Chinese government launched the first round of the NVBP in 11 pilot cities, later expanding it nationwide and establishing a periodic pooled procurement system. The first NVBP covered 25 medicines, in which antihypertensive medicines represented the largest therapeutic category (7/25). These antihypertensive medicines covered four common pharmacological classes: calcium channel blocker (CCB), angiotensin-converting enzyme inhibitor (ACEI), angiotensin receptor blocker (ARB), and ARB/diuretic combination (Table S1 in the **Online Supplementary Document**). In the first NVBP, the manufacturer offering the lowest bidding price of each medicines won the bid. Six out of seven bid-winning NVBP antihypertensive medicines were generic, with the left one being originator. It was reported that compared to the lowest prices in previous year in pilot cities, the price reduction for these NVBP antihypertensive medicines ranged from 50–90% [8].

This study is conducted in Tianjin, one of the pilot cities. Located in northeastern China, Tianjin is one of four provincial-level cities directly governed by the central government. It is administratively divided into 16 districts – six central urban districts and 10 others that contain both urban and rural areas. In 2019, Tianjin had a resident population of 15.6 million, comparable to a medium-sized European country, with 83.5% living in urban areas. Tianjin’s per capita disposable income reached 42 404 Chinese Yuan (CNY) in 2019, 140% of the national average. The first NVBP was officially implemented in Tianjin on 1 April 2019. Following the policy's implementation, health care institutions primarily procured the 25 medicines from the bid-winning manufactures (over 60% volume) [11,25].

### Data and study population

This retrospective cohort study utilised anonymous claims data from the Urban Employee Basic Medical Insurance (UEBMI) scheme in Tianjin, China, during 2017–2020. The UEBMI scheme is one of the two basic health insurance systems in China, which together with the Urban-Rural Resident Basic Medical Insurance, has achieved over 95% population coverage nationwide. The UEBMI mandates enrolment for all employees and retirees from both urban and rural areas. In Tianjin, UEBMI enrolees accounted for 52.3% of all basic medical insurance beneficiaries in 2019, and there were 5.9 million registered beneficiaries in this health system in 2019, of which 1.6 million were diagnosed with hypertension. A 30% random sample of beneficiaries were selected based on unique anonymous IDs assigned to each beneficiary in this health system. The database encompasses all medical and pharmacy claims from health care institutions and pharmacies for UEBMI beneficiaries, and is routinely subjected to administrative and financial audits by the health insurance authority, ensuring its accuracy and completeness.

**Figure 1:**
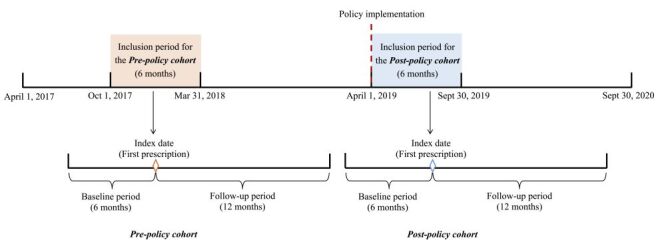
Study time design.

New-user study design was adopted by this study. Adults with a claim for at least one of the seven NVBP antihypertensive medicines during the pre-policy inclusion period (October 2017–March 2018) or the post-policy inclusion period (April–September 2019) were first identified, with their first claim defined as the ‘index’ claim (**Figure 1**). ‘New users’ were then defined as individuals without any antihypertensive medicine claims during the six-month baseline period preceding the index date (the date of the index claim). To enhance internal validity, we included only new users and excluded all prevalent users. This design preserves the appropriate temporal ordering of baseline confounders, treatment exposure, and outcomes, thereby minimising the risk of adjusting for variables influenced by prior treatment and reducing the potential for reverse causation. This approach is also supported by prior methodological literature [26–28]. New users with index date in the post-policy inclusion period formed the ‘post-policy cohort’, while those with index date in the pre-policy inclusion period constituted the ‘pre-policy cohort’.

Each cohort were followed for one year since the index date. We further excluded patients who discontinued enrolling the UEBMI during the follow-up period (except for those who discontinued enrolment due to death) to ensure the completeness of their claim records. Those with only one antihypertensive medicine claim during the follow-up period were also excluded, as they were likely occasional users. Two confounding policies were implemented in Tianjin during the study period: the second round NVBP policy on 25 April 25 2024, covering five antihypertensive medicines (olmesartan, candesartan, bisoprolol, trazodone, indapamide), and the third round on 1 November 2020, covering two antihypertensive medicines (captopril, valsartan). To mitigate potential confounding from subsequent NVBP rounds and maintain comparability between cohorts, we excluded patients in both cohorts who had prescription claims for any of these seven additional antihypertensive medicines during the follow-up.

### Outcomes

#### Adherence

We used the proportion of days covered (PDC) to ascertain patient adherence to medicines during the follow-up period, which was calculated as the number of days covered by the medicine prescriptions divided by the total number of follow-up days (365 days). We also measured patient persistence to medicines, using the indicator of discontinuation rates, defined as having a gap of more than 90 days since the end date of the last prescription, regardless of whether the therapy was resumed thereafter [29,30]. Overlapping days between medicine prescriptions were only counted once when calculating PDC and discontinuation rates. We first estimate patients’ adherence and persistence to NVBP antihypertensive medicines. For those who used other antihypertensive medicines during the one-year follow-up, we also reported their adherence and persistence outcomes to these medicines separately.

#### Costs

We measure patients’ annual total direct medical costs during the follow-up period. Our primary focus was on the hypertension-related costs, defined as costs associated with outpatient or inpatient claims with a diagnosis of hypertension. We further analysed costs for medicines in hypertension-related costs, with particular emphasis on the costs for NVBP antihypertensive medicines. Meanwhile, we break down hypertension-related costs by payer, including costs on the health plan and patient out-of-pocket costs. Non-hypertension-related costs, defined as expenses linked to outpatient or inpatient claims without any hypertension diagnosis, were also examined. The sum of hypertension-related and non-hypertension-related costs constituted the all-cause costs.

### Covariates

We adjust three types of baseline characteristics. For demographic characteristics, we include age, sex (male/female), whether the patients were retired, and the types of their last working institutions (government organs and public institutions/ company/ others). For medical history, we include the Charlson comorbidity index (CCI), dyslipidaemia, myocardial infarction, stroke, heart failure, atrial fibrillation, diabetes, and chronic kidney disease, as suggested by guideline for hypertension management [24]. We also include patients’ baseline health care utilisation characteristics, including admission and the number of outpatient visits for any cause. These covariates were selected based on their potential association with both the post-policy use of NVBP medicines and the study outcomes, as supported by previous studies or clinical guidelines [17,24,31,32].

### Statistical analysis

We report the mean (standard deviations, SD) and frequencies (proportions) of patients’ baseline characteristics for each cohort, and adopt χ^2^ tests and *t* test to compare between cohorts. We adopt linear regressions to compare adherence differences, Cox proportional hazard models to compare persistence, and logit regressions to compare the probability of using non-NVBP antihypertensive medicines between cohorts. General linear models with logarithmic link function and gamma distribution were employed to estimate cost differences between cohorts. All regression models were adjusted for the full set of baseline covariates mentioned above. Standard errors were clustered at the hospital level based on where patients received their index NVBP antihypertensive prescription to account for potential within-hospital correlation. Bar charts and forest plots were used to compare outcome differences between cohorts. The significance level was set at two-sided α<0.05. Statistics analysis was conducted using STATA 17.0 (StataCorp LP, College Station, Texas, USA).

#### Sensitivity analyses

First, we evaluate whether the policy effects varied across age groups, stratifying the cohorts into the elderly (≥60 years) and non-elderly (<60 years) subgroups. Second, we examine the policy effects across male and female subgroups. Interaction term between the cohort and subgroup were included in the regression models, with Wald tests used to assess difference in policy effects between subgroups. Third, given that clinical practices, resource availability, and prescribing behaviours often vary across different levels of health care institutions in China [33], which may in turn influence patients’ outcomes, we conducted subgroup analysis by the level of health care institution (*i.e.* primary, secondary, or tertiary) where patients received their index antihypertensive prescription. Forth, the seven NVBP antihypertensive medicines are classified into four pharmacological subgroups based on their mechanisms of action: CCB (amlodipine), ACEI (fosinopril, lisinopril, enalapril), ARB (irbesartan, losartan), and ARB/diuretic combination (irbesartan-hydrochlorothiazide). To investigate whether the policy impact varies across users of medicines from different pharmacological subgroups, we classified patients into four subgroups based on the pharmacological category of their index prescription. Patients spanning multiple subgroups were excluded from the analysis. Fifth, we employ propensity-score matching to create comparable pre- and post-policy cohort pair for the main analyses. Propensity scores were estimated using logistic regressions, with all baseline covariates included, and we then perform 1:1 nearest matching without replacement were performed. For the matched pre- and post-policy cohort pairs, we conducted univariate regression analyses to compare differences in outcome measures.

## RESULTS

A total of 183 669 and 211 580 adult patients with at least one claim for NVBP antihypertensive medicines were identified during the post-policy and pre-policy inclusion periods, respectively. Among them, 37 796 and 43 272 ‘new users’ were included in the post-policy and pre-policy cohorts, respectively. Based on the follow-up period selection criteria, for the post-policy (pre-policy) cohort, we further excluded 2421 (5874) patients who discontinued UEBMI enrolment, 11 660 (19 470) patients with only one antihypertensive prescription claim, and 4857 (3368) patients who used the second- or third-round NVBP antihypertensive medicines. The final sample sizes were 18 858 in the post-policy cohort and 14 560 in the pre-policy cohort, with no overlap between them (Figure S1 in the **Online Supplementary Document**).

### Patient characteristics

The mean age of the two cohorts was 57.1 years (SD = 14.0), with 52.8% being male (**Table 1**). Most patients were employed or previously employed in companies (77.7%), followed by government organs and public institutions (22.0%). The mean CCI = 0.74 (SD = 1.15), with 20.6, 15.8, and 4.7% having records of dyslipidaemia, diabetes, and chronic kidney diseases, respectively. The proportion of patients with baseline cardiovascular disease records were small, with 2.0% having a history of stroke, 0.5% heart failure, 0.4% atrial fibrillation, and 0.3% myocardial infarction. A total of 8.8% of patients were hospitalised during the baseline period.

**Table 1:** Baseline characteristics of the patients in the pre-policy and post-policy cohorts

Characteristics	Total (N = 33 418)	Pre-policy cohort (N = 14 560)	Post-policy cohort (N = 18 858)	*P*-value
Age, x̄ (SD), years	57.1 (14.0)	56.3 (14.3)	57.7 (13.7)	<0.0001
Age group, n (%), years				<0.0001
*≤19*	0 (0)	0 (0)	0 (0)	
*20–29*	370 (1.1)	238 (1.6)	132 (0.7)	
*30–39*	3850 (11.5)	1912 (13.1)	1938 (10.3)	
*40–49*	6355 (19.0)	2761 (19.0)	3594 (19.1)	
*50–59*	7847 (23.5)	3347 (23.0)	4500 (23.9)	
*60–69*	8549 (25.6)	3657 (25.1)	4892 (25.9)	
*70–79*	4485 (13.4)	1796 (12.3)	2689 (14.3)	
*≥80*	1962 (5.9)	849 (5.8)	1113 (5.9)	
Male, n (%)	17 642 (52.8)	7623 (52.4)	10 019 (53.1)	0.161
Job, n (%)				<0.0001
*Working*	17 109 (51.2)	7330 (50.3)	9779 (51.9)	
*Retired*	16 309 (48.8)	7230 (49.7)	9079 (48.1)	
Institution, n (%)				0.009
*Government organs and public institutions*	7335 (22.0)	3148 (21.6)	4187 (22.20)	
*Company*	25 964 (77.7)	11 375 (78.1)	14 589 (77.4)	
*Others*	119 (0.4)	37 (0.3)	82 (0.4)	
Medical history, n (%)				
*CCI, x̄ (SD)*	0.74 (1.15)	0.75 (1.16)	0.75 (1.13)	0.593
*Myocardial infarction*	82 (0.3)	15 (0.1)	67 (0.4)	<0.0001
*Stroke*	668 (2.0)	274 (1.9)	394 (2.1)	0.179
*Heart failure*	173 (0.5)	85 (0.6)	88 (0.5)	0.139
*Atrial fibrillation*	129 (0.4)	59 (0.4)	70 (0.4)	0.619
*Dyslipidaemia*	6891 (20.6)	2959 (20.3)	3932 (20.9)	0.237
*Diabetes*	5286 (15.8)	2382 (16.4)	2904 (15.4)	0.017
*Chronic kidney disease*	1556 (4.7)	685 (4.7)	871 (4.6)	0.712
Baseline health care utilisation				
*Admissions, n (%)*	2954 (8.8)	1257 (8.6)	1697 (9.0)	0.106
*Number of outpatient visits, x̄ (SD)*	28.2 (16.9)	28.5 (16.7)	28.1 (18.2)	0.072

CCI – charlson comorbidity index, n –number, SD – standard deviation, x̄ – mean

New-users in the pre- and post-policy cohorts were largely comparable, with few exceptions. Patients in the post-policy cohort were slightly older than those in the pre-policy cohort (57.7 *vs*. 56.3 years, *P* < 0.0001), had a lower proportion of patients employed in companies (77.4 *vs*. 78.1%, *P* = 0.009), a higher prevalence of baseline myocardial infarction (0.4 *vs*. 0.1%, *P* < 0.0001).

### Adherence

**Figure 2:**
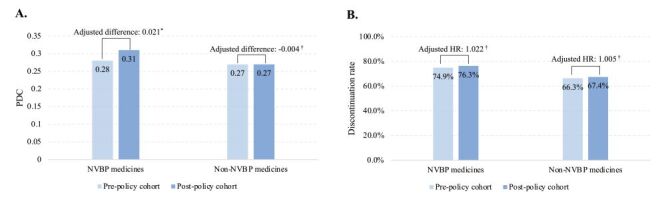
Comparison of the adherence to and persistent on antihypertensive medicines during one-year follow-up period between the pre-policy and post-policy cohorts. **Panel. A.** Adherence. **Panel B.** Persistence. *Indicates *P* < 0.05. †Indicates *P* ≥ 0.05. HR – hazard ratio, PDC – proportion of days covered.

Over the one-year follow-up period, adherence to the NVBP antihypertensive medicines was 0.31 (SD = 0.22) in the post-policy cohort and 0.28 (SD = 0.22) in the pre-policy cohort (**Figure 2**). Linear regressions estimated a significant difference of 0.021 (95% confidence interval (CI) = 0.016, 0.025; *P* < 0.0001). The discontinuation rate for NVBP antihypertensive medicines was 74.9% in the pre-policy cohort and 76.4% in the post-policy cohort, showing no statistically significant difference (hazard ratio (HR) = 1.022; 95% CI = 0.894, 1.102, *P* = 0.506). However, fewer patients in the post-policy cohort had used non-NVBP antihypertensive medicines during the follow-up (58.4 vs 66.6%), with a 37.1% lower probability compared to the pre-policy cohort (95% CI = −41.7%, −32.6%, *P* < 0.0001). Among patients who used non-NVBP antihypertensive medicines, the mean PDC and discontinuation rates for these medicines did not differ significantly between cohorts.

### Costs

**Figure 3:**
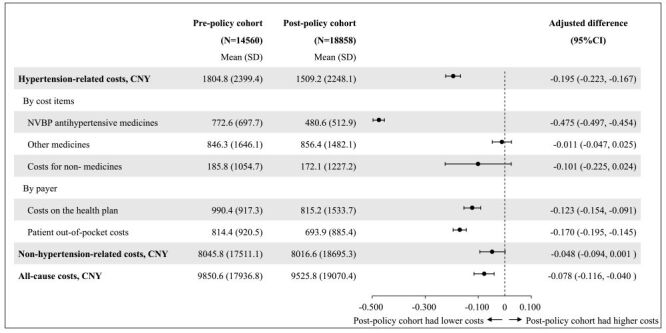
Comparison of costs during one-year follow-up period between the pre-policy and post-policy cohorts.

Patients in the post-policy cohort incurred annual all-cause costs of 9525.8 CNY (SD = 19 070.4), 7.8% (95% CI = −11.6%, −4.0%, *P* < 0.0001) lower than patients in the pre-policy cohort (**Figure 3**). This cost difference was entirely attributed to saving in hypertension-related costs (1509.2 *vs*. 1804.8 CNY; relative difference = −19.5%; 95% CI = −22.3%, −16.7%, *P* < 0.0001), as there was no significant difference in non-hypertension-related costs between cohorts (8016.6 *vs*. 8045.8 CNY; relative difference = −4.8%; 95% CI = −9.4%, 0.1%, *P* = 0.052). Among hypertension-related costs, the annual costs for NVBP antihypertensive medicines were 480.6 CNY (SD = 512.9) in the post-policy cohort and 772.6 CNY (SD = 697.7) in the pre-policy cohorts, representing a 47.5% reduction (95% CI = −49.7%, −45.4%, *P* < 0.0001) in the post-policy cohort. No significant differences were observed in costs for other medicines between cohorts. The cost savings for the post-policy cohort were similarly distributed between the health plan and the enrolees, each accounting for approximately 12–17%.

### Sensitivity analyses

The policy effects on adherence and costs are consistent between males and females (Figure S2–3 and Table S2 in the **Online Supplementary Document**), as nearly all interaction terms were insignificant. Whereas, the policy impact varies across age subgroups (Figure S2, S4 and Table S3 in the **Online Supplementary Document**). Compared to patients under 60 years old, the policy effect on cost saving is significantly greater in patients over 60 (*e.g*. costs for NVBP antihypertensive medicines: interaction coefficient = −11.0%; 95% CI = −15.3%, −6.7%, *P* < 0.0001). In both pre- and post-policy cohorts, most patients received their index antihypertensive prescription in primary health care institutions (pre-policy cohort = 65.2%; post-policy cohort = 68.3%), followed by secondary (pre-policy cohort = 14.0%; post-policy cohort = 12.7%) and tertiary institutions (pre-policy cohort = 20.7%; post-policy cohort = 19.1%). Patients in the subgroup of tertiary institutions had higher medical costs. For example, in the post-policy cohort, the average hypertension-related costs were 1350.2, 1441.2 and 2123.4 CNY in primary, secondary, and tertiary institutions, respectively. We also observed larger cost-saving for patients in the subgroup of tertiary institutions (Figure S2, S5 and Table S4 in the **Online Supplementary Document**). The results of subgroup analyses, classifying patients according to the pharmacological classes of their index prescription (*i.e*. CCB, ARB, ACEI, ARB/diuretic combination), mostly align with the main analyses (Figure S2, S6 and Table S5 in the **Online Supplementary Document**). Finally, adopting propensity-score matching for the main analyses (Figure S2, S7 and Table S6–8 in the **Online Supplementary Document**) provides consistent findings too.

## DISCUSSION

### Main findings

Since 2019, Chinese government has implemented the nationwide pooled procurement for medicines, the NVBP, to reduce medicine prices and alleviate economic burden of patients and health plans. Focusing on new users of NVBP antihypertensive medicines, we find that the NVBP in China successfully reduced the medical costs for the patients and health plan, by saving costs from the NVBP medicines, without affecting other costs. Adherence to NVBP medicines was improved after the policy, although the improvement was modest. The cost saving effects were more profound in elderly patients and patients who visited tertiary health care institutions.

### Interpretations

We found that pooled procurement improved patient adherence to antihypertensive medicines covered by the policy. This aligns with a recent study that focused on patients who underwent generic substitutions after the NVBP, although that study focused on a single medicine [17]. This finding is expected, as prior research has shown that reducing medicine prices or patient cost-sharing improves medicine affordability and addresses unmet treatment needs, leading to increased medication use [13–16]. Earlier evaluation of the pooled procurement in China and other countries also documented substantial procurement volume increases alongside price reductions [11,12,34,35], indirectly supporting our findings.

However, the improvement in adherence was modest, with the post-policy cohort showing only a 0.021 (approximately 10%) increase in PDC compared to the pre-policy cohort; discontinuation rates also remained high and similar between cohorts. This may result from the generally poor adherence/persistence among hypertensive patients in China [17,36–38], leaving limited room for improvement. Moreover, while NVBP reduced medicine prices, it did not address other major drivers of adherence/persistence, such as factors related to health care system (*e.g.* physician’s communication style), patient (*e.g*. perception of illness) and therapy (*e.g*. choice of complex regimens) [32,39]. As a result, its impact on improving adherence and persistence may be limited. Previous studies have consistently shown that better adherence to antihypertensive medicines is associated with improved clinical outcomes, mostly by comparing patients with PDC over 0.8 to those below 0.8 [40,41]. However, in our study, only about 4% of patients in either cohort reached this threshold (data not shown). As such, it remains unclear whether the modest improvement in adherence observed here is sufficient to yield clinical benefits, which warrants further long-term investigation.

Despite increased consumption of NVBP medicines, patients still saved nearly 50% on NVBP medicine costs over the one-year follow-up period, as price reduction outweighed the increased usage. This trend aligns with institutional-level evidence from countries such as Brazil, Colombia, New Zealand and China [7,11,12,42,43], where pooled procurement has significantly reduced procurement expenditure of medicines. Although patient-level studies remain limited, previous evidence also show declines in per-visit outpatient costs after NVBP implementation [21,23], without distinguishing between types of costs.

This study also shows that the NVBP policy effectively reduced patients' financial burden by specifically lowering costs of NVBP medication, without shifting costs to other medicine or non- medicine expenses. In contrast, numerous studies across countries have indicated that cost-shifting is very common after the implementation of pricing policy, as health care providers may seek to offset revenue losses from price adjustments [44–51]. No cost-shifting was observed after NVBP in this study, likely due to the distinctive policy design on both the supply and demand sides of China’s pooled procurement system. On the supply side, regulatory enforcement and financial incentives jointly discouraged providers from shifting costs. First, health care institutions were required to fulfil substantial purchasing volume commitment (60 ~ 80% of the previous year's total usage volume for the same generic molecule) and to prioritise the use of bid-winning medicines in clinical practice, with regular oversight by local medical insurance bureaus. Second, the National Healthcare Security Administration issued lists of ‘therapeutic alternative medicines’ to the bid-winning medicines and urged local authorities to closely monitor their procurement and use to avoid unnecessary therapeutic switching. Third, health care institutions in Tianjin that met procurement targets could receive financial rewards from health insurance funds, while noncompliant ones faced audits or penalties. On the demand side, besides lowering prices, the policy promoted patient acceptance of bid-winning medicines by setting the reimbursement rate for bid-winning medicines 10% higher than non-winning ones, reducing patients’ out-of-pocket costs. In addition, the government launched widespread public campaigns to build trust in the safety and efficacy of bid-winning medicines, mitigating resistance to usage. Although no cost-shifting was observed in this study, it may still occur under NVBP. The analysis focused only on the first pilot NVBP round, when provider responses could have been delayed. The one-year follow-up also makes it difficult to speculate longer-term effects.

Although the subgroup analyses and sensitivity analyses yielded results consistent with the main findings, it is noteworthy that cost-saving effects were significantly greater among patients over 60 years. This may be explained by their greater disease severity (baseline CCI = 0.80 *vs*. 0.72; data not shown) and thus higher consumption of NVBP medicines during follow-up (PDC of NVBP medicines = 0.33 *vs*. 0.27, data not shown), compared with those under 60 years, making elderly patients more likely to benefit from price reductions of NVBP medicines. A similar trend was observed among patients in the subgroup of tertiary institutions, which may be attributable to similar reasons.

### Policy implications

Our findings have two policy implications. First, although NVBP significantly reduced medicine prices (by 50–90%) [8], it led to only slight improvements in patient adherence. For chronic conditions like hypertension, adherence is a key determinant of health outcomes [52,53]. Given the global challenge of poor adherence among chronic disease patients [54], pooled procurement of chronic disease medicines should be accompanied with broader supportive strategies to improve treatment compliance, such as provider training and patient education, thereby promoting systematic care and reducing overall medical costs. For acute or short-term conditions, efforts may instead focus on timely access and supply continuity. Second, our findings show that NVBP effectively reduced patients' NVBP medicine costs without cost-shifting, highlighting its potential to ease financial burden for both patients and health care systems. To achieve this, patients must have access to the bid-winning medicines and receive appropriate treatment from providers. Policymakers should assess how pooled procurement may affect provider incentives beforehand and design auxiliary supply- and demand-side measures to support appropriate provider and patient behaviour. The experience in Tianjin, as discussed above, may offer useful insights. Long-term monitoring is also critical to detect and prevent unintended consequences, such as substitution effects.

### Strengths and limitations

We conduct the first cohort study to investigate the impact of pooled procurement on medical costs from the perspective of patients, with all NVBP medicines from the same therapeutic class included. By adopting the new-user design, we minimise the selection bias and provide clear and valid evidences to better understand the policy effects on patients.

This study has several limitations. First, the sample was limited to UEBMI enrolees in Tianjin, China, which may affect generalisability. The UEBMI scheme covers only employees and retirees, while informal-sector workers and non-employed are enrolled in Urban-Rural Resident Basic Medical Insurance. In addition, Tianjin is relatively high-urbanised, with 83.5% of its population resides in urban area, limiting extrapolation to rural settings. Second, we focused on new users of antihypertensive medicines, which may limit the generalisability to prevalent users who might exhibit different adherence and cost patterns [55,56]. We also excluded patients who used antihypertensive medicines included in later NVBP rounds. Although this affected a small proportion (<13%), this may slightly limit the generalisability. Third, our study restricted to patients with hypertension, while NVBP also covers other chronic conditions (*e.g*. diabetes) and acute conditions (*e.g*. bacterial infections). Although antihypertensive medicines are representative within NVBP, caution is warranted when generalising these findings. Fourth, adherence was measured using PDC based on prescription claims, which reflects dispensing, not actual intake, and may overestimate adherence. Fifth, the one-year follow-up period limits our ability to assess long-term effects. In addition, given the low incidence of mortality and cardiovascular events (less than 3%; data not shown) among new users during this relatively short follow-up, and the limitations of claims data in identifying mild or uncoded side effects, the clinical outcomes were not also assessed. Sixth, while we adjusted for observable confounders (*i.e*. baseline characteristics), residual confounding (*e.g*. blood pressure levels, smoking) and unrelated external shocks may remain. However, the new-user design likely reduces such bias, as the characteristics of new cases of chronic diseases tend to remain stable over short periods, supported by the balanced baseline characteristics between cohorts in our study. Also, no major health policy changes occurred in Tianjin during the study period. Lastly, regional heterogeneity could not be assessed due to missing geographic information, though such variation is likely minimal in Tianjin given its small size, unified health system, and relatively even health care resource distribution.

## CONCLUSIONS

China’s nationwide pooled procurement of medicines, the NVBP, slightly improved adherence for new-users of antihypertensive medicines and alleviated their economic burden by reducing the costs of targeted medicines. These findings suggest that the price reduction is only the first step toward effective pooled procurement for chronic disease medicines. To fully realise patient-centred benefits of pooled procurement, auxiliary supply- and demand-side measures – such as provider training and patient education, provider incentives and oversight, and favourable reimbursement policies – are essential to improve adherence and regulate provider behaviour to prevent cost-shifting. Future research should conduct longer-term evaluations in broader populations to better understand the policy’s overall impact.

## Additional material


Online Supplementary Document
