# Supplementary material for: Impact of pooled procurement of medicines on patient adherence and economic burden: evidence from China
Source: J Glob Health. 2025 Aug 22;15:04229. doi: 10.7189/jogh.15.04229 (PMC12371299; doi:10.7189/jogh.15.04229)
Supplement: Online Supplementary Document [file jogh-15-04229-s001.pdf]

Supplement to: Zhao B, Wu J, Cheng Z, Feng XL. Impact of pooled procurement of medicines on patient adherence and economic burden: evidence from China. J Glob Health. 2025;15:04229.

Table of Contents

Figure S1. Sample selection flowchart 1

Figure S2. Subgroup analyses on difference in key outcome variables: adherence to and costs for NVBP antihypertension medicines. 2

Figure S3. Subgroup analyses by sex: Comparison of the adherence to and persistent on antihypertensive medicines during one-year follow-up period between the pre-policy and post-policy cohorts 3

Figure S4. Subgroup analyses by age: Comparison of the adherence to and persistent on antihypertensive medicines during one-year follow-up period between the pre-policy and post-policy cohorts 4

Figure S5. Subgroup analyses by levels of healthcare institutions: Comparison of the adherence to and persistent on antihypertensive medicines during one-year follow-up period between the pre-policy and post-policy cohorts 5

Figure S6. Subgroup analyses by types of index medicines: Comparison of the adherence to and persistent on antihypertensive medicines during one-year follow-up period between the pre-policy and post-policy cohorts 6

Figure S7. Sensitivity analyses: Comparison of the adherence to and persistent on antihypertensive medicines during one-year follow-up period between the pre-policy and post-policy cohorts, after propensity score matching 7

Table S1. Information about bid-winning antihypertensive medicines in the pilot NVBP in China. 9

Table S2. Subgroup analyses by sex: Comparison of the costs during one-year follow-up period between the pre-policy and post-policy cohorts 10

Table S3. Subgroup analyses by age: Comparison of the costs during one-year follow-up period between the pre-policy and post-policy cohorts 11

Table S4. Subgroup analyses by levels of healthcare institutions: Comparison of the costs during one-year follow-up period between the pre-policy and post-policy cohorts 14

Table S5. Subgroup analyses by types of index medicines: Comparison of the medical costs during one-year follow-up period between the pre-policy and post-policy cohorts 15

Table S6. Sensitivity analyses: Baseline characteristics of the patients in the pre-policy and post-policy cohorts, after propensity score matching 17

Table S7. Sensitivity analyses: Comparison of the adherence to antihypertensive medicines during one-year follow-up period between the matched pre-policy and post-policy cohorts for the main analysis, after propensity score matching 18

Table S8. Sensitivity analyses: Comparison of the costs during one-year follow-up period between the matched pre-policy and post-policy cohorts for the main analysis, after propensity score matching 20

Checklist S1. STROBE Statement—Checklist of items that should be included in reports of cohort studies 21

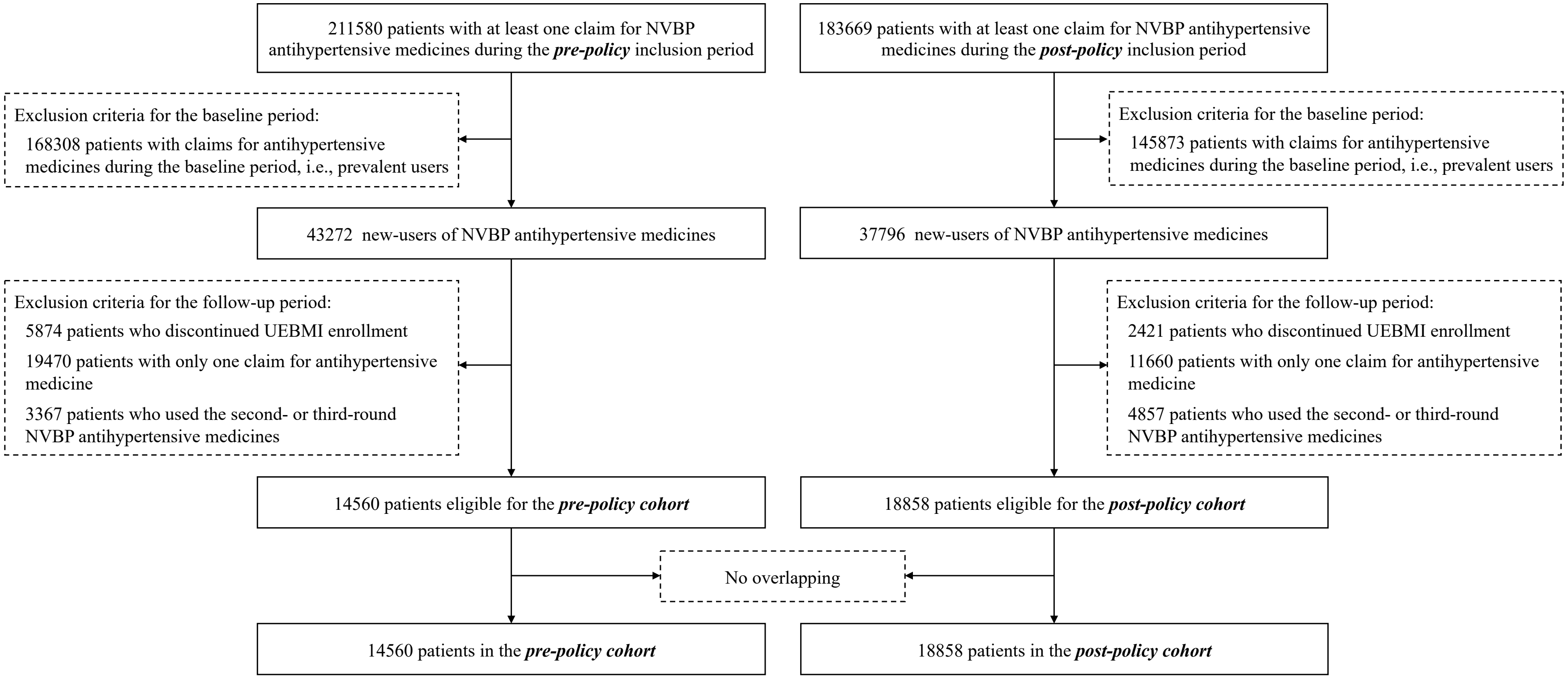

Figure S1. Sample selection flowchart

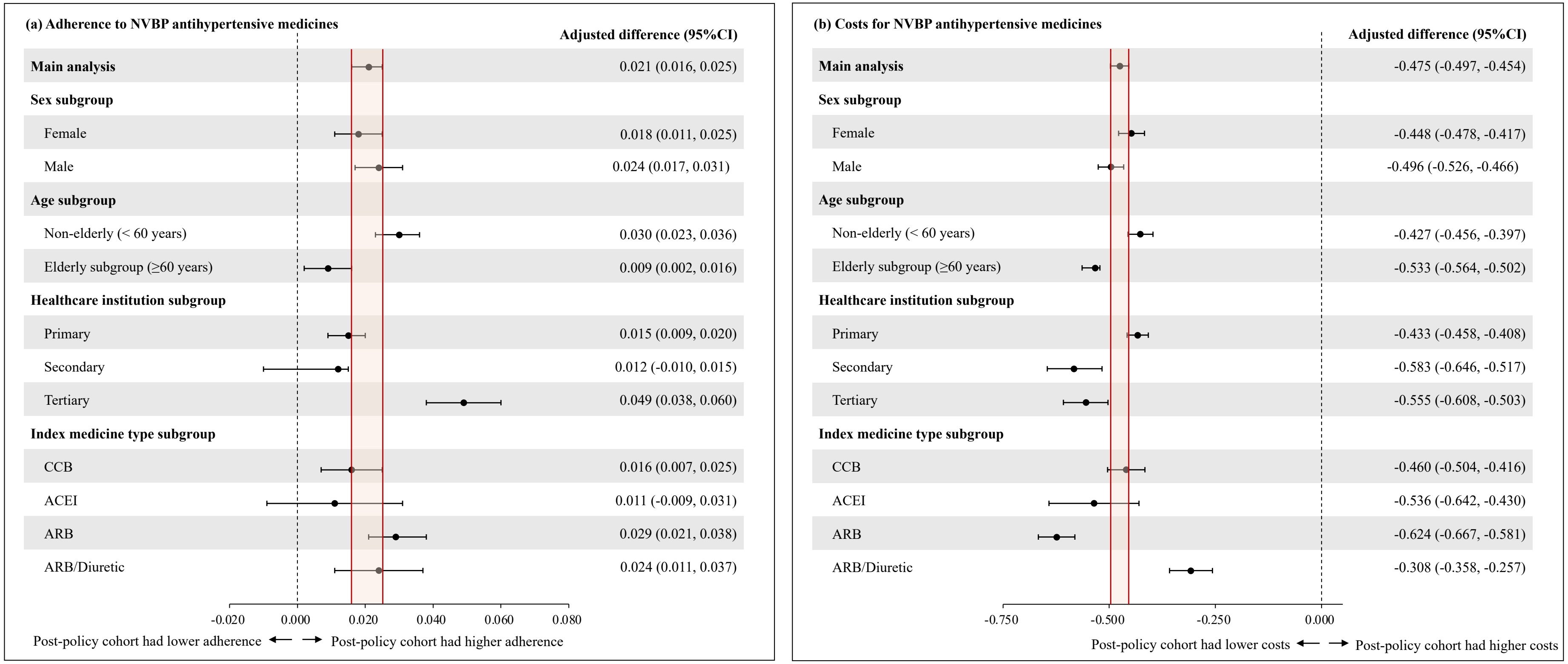

Figure S2. Subgroup analyses on difference in key outcome variables: adherence to and costs for NVBP antihypertension medicines.

The differences in adherence and costs between cohorts were estimated using linear regressions and general linear models (GLM) with logarithmic link function and gamma distribution, respectively, with adjustments for all baseline covariates.

CCB - calcium channel blocker, ACEI - angiotensin-converting enzyme inhibitor, ARB - angiotensin receptor blocker.

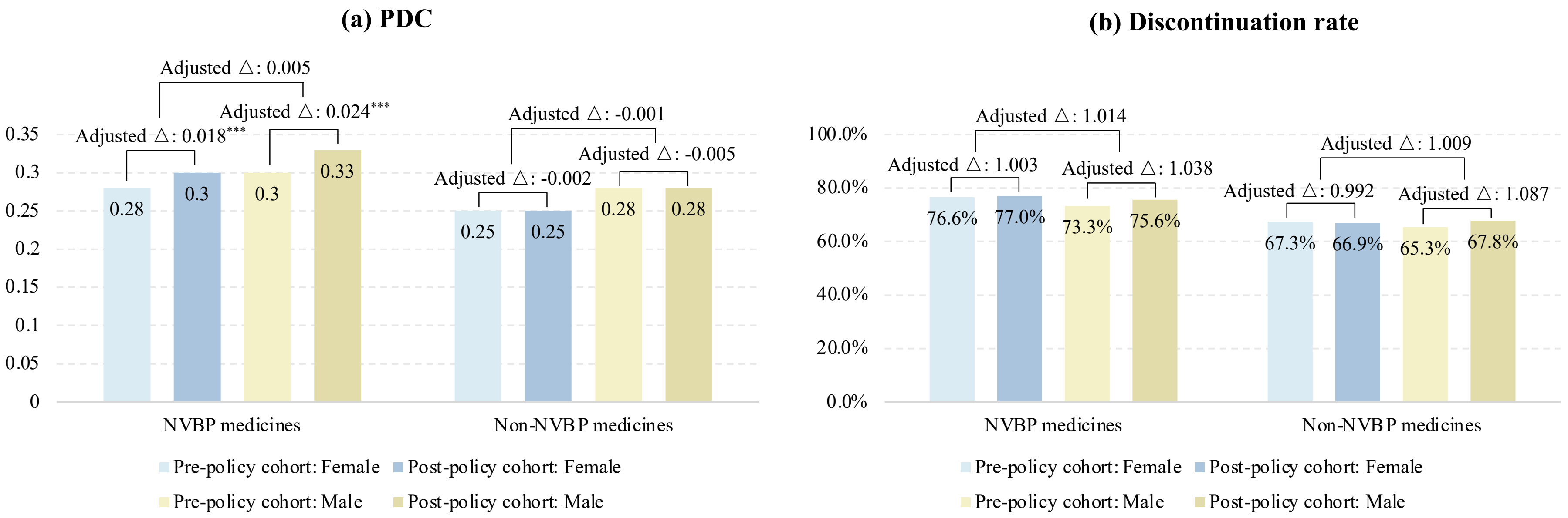

Figure S3. Subgroup analyses by sex: Comparison of the adherence to and persistent on antihypertensive medicines during one-year follow-up period between the pre-policy and post-policy cohorts

* Indicates $p < 0.05$, ** Indicates $p < 0.01$, *** Indicates $p < 0.001$. PDC, proportion of days covered. △, difference.

Sample sizes: Female: pre-policy cohort: 6937 patients, post-policy cohort: 8839 patients; Male: pre-policy cohort: 7623 patients, post-policy cohort: 10019 patients.

The PDC and discontinuation rate of non-NVBP antihypertensive medicines were estimated for patients who used non-NVBP antihypertensive drugs during the one-year follow-up (Female: pre-policy cohort: 4529 patients, post-policy cohort: 5136 patients; Male: pre-policy cohort: 5173 patients, post-policy cohort: 5875 patients).

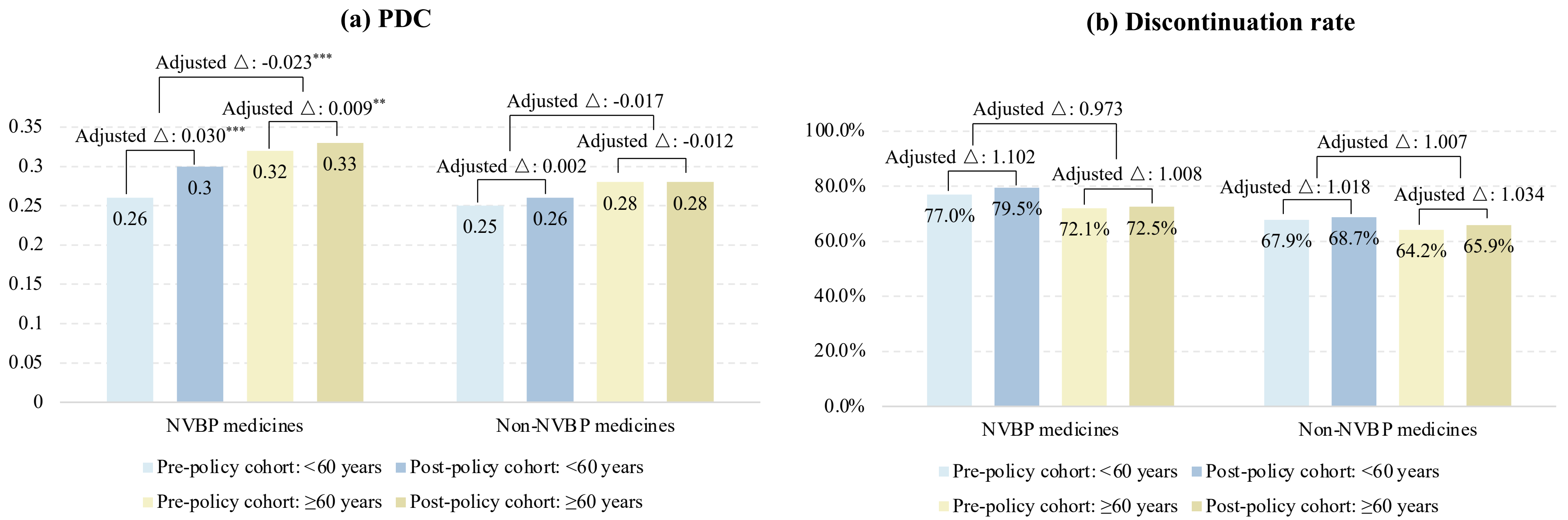

Figure S4. Subgroup analyses by age: Comparison of the adherence to and persistent on antihypertensive medicines during one-year follow-up period between the pre-policy and post-policy cohorts

* Indicates $p < 0.05$, ** Indicates $p < 0.01$, *** Indicates $p < 0.001$. PDC, proportion of days covered. △, difference.

Sample size: Non-elderly (<60 years): pre-policy cohort: 8258 patients, post-policy cohort: 10164 patients; Elderly (≥60 year): pre-policy cohort: 6302 patients, post-policy cohort: 8694 patients.

The PDC and discontinuation rate of non-NVBP antihypertensive medicines were estimated for patients who used non-NVBP antihypertensive drugs during the one-year follow-up (Non-elderly: pre-policy cohort: 5350 patients, post-policy cohort: 5836 patients; Elderly: pre-policy cohort: 4352 patients, post-policy cohort: 5175 patients).

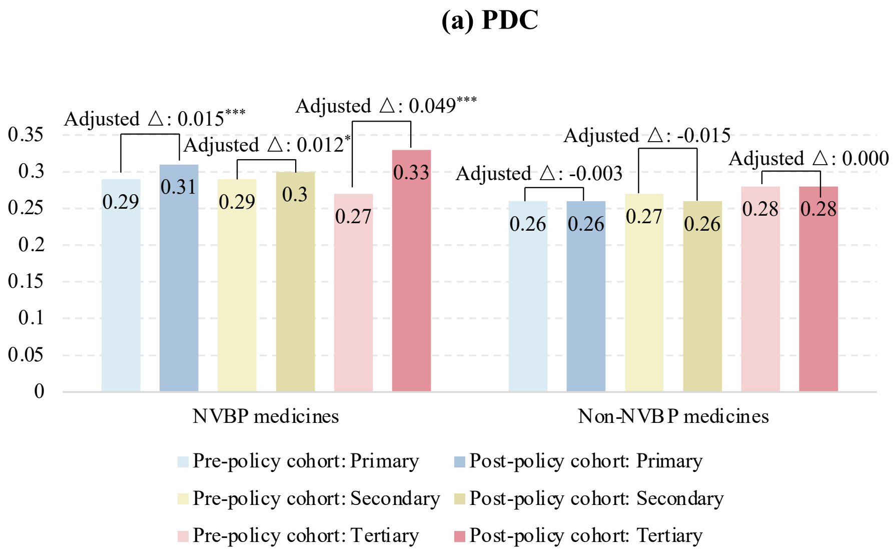

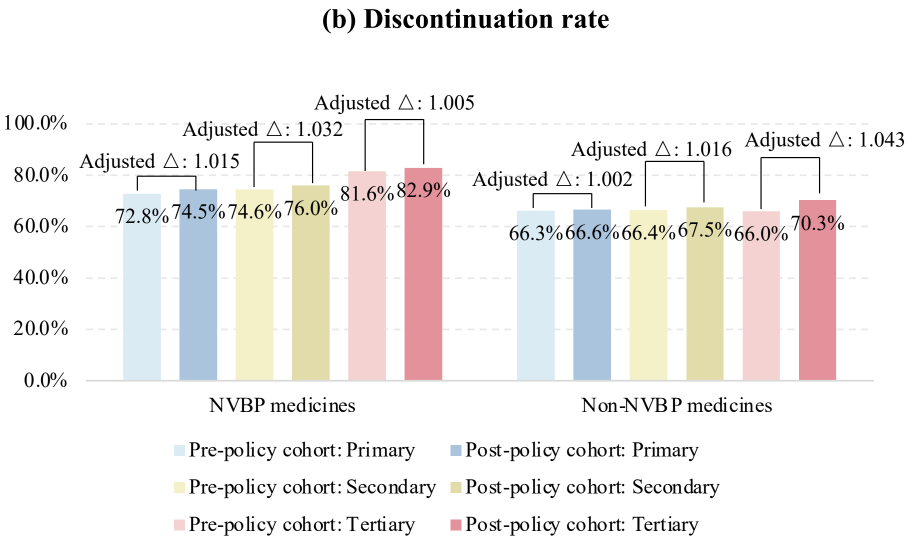

Figure S5. Subgroup analyses by levels of healthcare institutions: Comparison of the adherence to and persistent on antihypertensive medicines during one-year follow-up period between the pre-policy and post-policy cohorts

* Indicates $p < 0.05$, ** Indicates $p < 0.01$, *** Indicates $p < 0.001$. PDC, proportion of days covered. △, difference.

Sample sizes: Primary: pre-policy cohort: 9498 patients, post-policy cohort: 12875 patients; Secondary: pre-policy cohort: 2044 patients, post-policy cohort: 2387 patients; Tertiary: pre-policy cohort: 3018 patients, post-policy cohort: 3596 patients.

The PDC and discontinuation rate of non-NVBP antihypertensive medicines were estimated for patients who used non-NVBP antihypertensive drugs during the one-year follow-up (Primary: pre-policy cohort: 6352 patients, post-policy cohort: 7547 patients; Secondary: pre-policy cohort: 1375 patients, post-policy cohort: 1405 patients; Tertiary: pre-policy cohort: 1975 patients, post-policy cohort: 2059 patients).

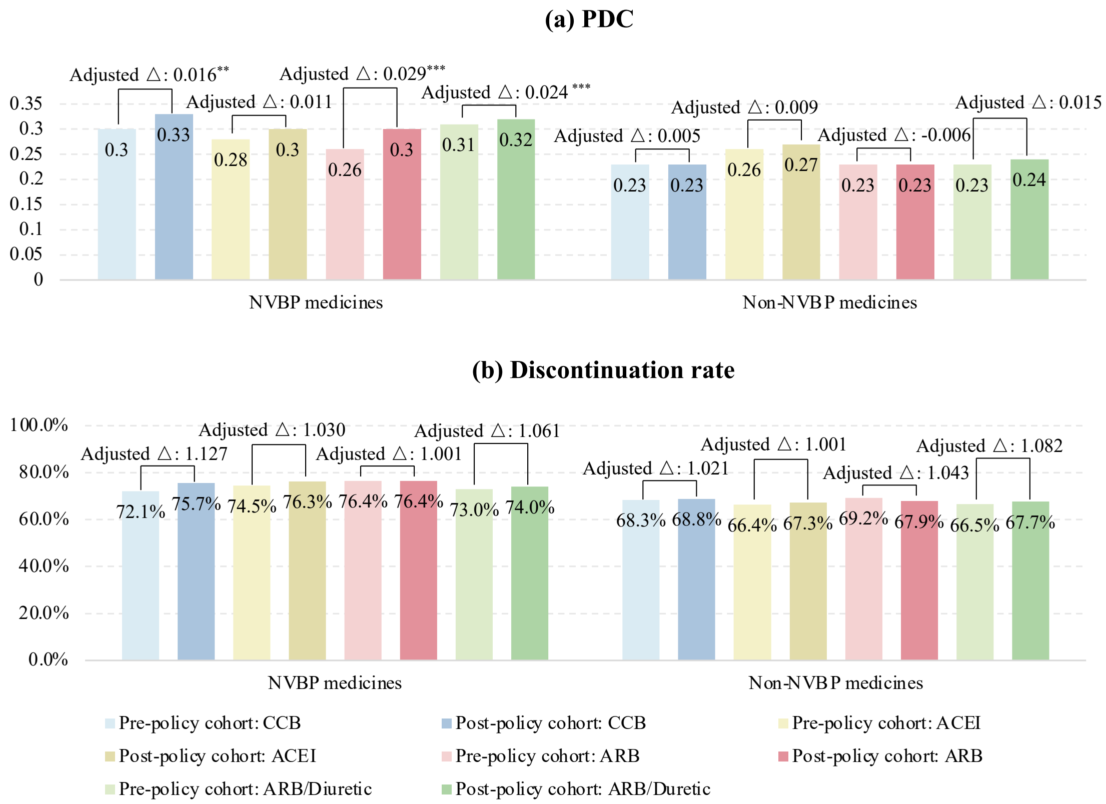

Figure S6. Subgroup analyses by types of index medicines: Comparison of the adherence to and persistent on antihypertensive medicines during one-year follow-up period between the pre-policy and post-policy cohorts

* Indicates $p < 0.05$, ** Indicates $p < 0.01$, *** Indicates $p < 0.001$. PDC, proportion of days covered. △, difference.

Sample sizes: CCB: pre-policy cohort: 3449 patients, post-policy cohort: 5688 patients; ACEI: pre-policy cohort: 737 patients, post-policy cohort: 908 patients; ARB: pre-policy cohort: 3972 patients, post-policy cohort: 4960 patients; ARB/Diuretic: pre-policy cohort: 1963 patients, post-policy cohort: 2395 patients. The PDC and discontinuation rate of non-NVBP antihypertensive medicines were estimated for patients who used non-NVBP antihypertensive drugs during the one-year follow-up (CCB: pre-policy cohort: 1865 patients, post-policy cohort: 2740 patients; ACEI: pre-policy cohort: 446 patients, post-policy cohort: 472 patients; ARB: pre-policy cohort: 2332 patients, post-policy cohort: 2411 patients; ARB/Diuretic: pre-policy cohort: 1239 patients, post-policy cohort: 1295 patients).

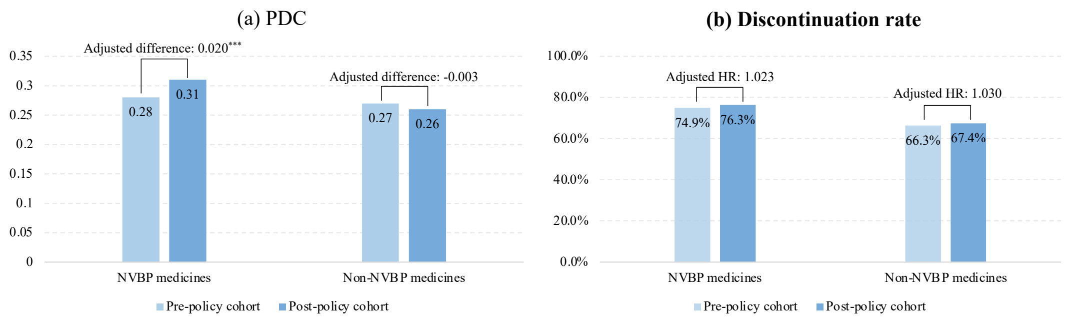

Figure S7. Sensitivity analyses: Comparison of the adherence to and persistent on antihypertensive medicines during one-year follow-up period between the pre-policy and post-policy cohorts, after propensity score matching

* Indicates $p < 0.05$, ** Indicates $p < 0.01$, *** Indicates $p < 0.001$. PDC, proportion of days covered. HR, hazard ratio.

Sample size: Pre-policy cohort: 14286 patients, Post-policy cohort: 14286 patients.

The PDC and discontinuation rate of non-NVBP antihypertensive medicines were estimated for patients who used non-NVBP antihypertensive drugs during the one-year follow-up (Pre-policy cohort: 9527 patients, Post-policy cohort: 8291 patients).

Table S1. Information about bid-winning antihypertensive medicines in the pilot NVBP in China.

Pharmacological classes	INN	Specification	Administration route /Form	Package size	Manufacturer	Original	Bid-winning price (CNY/package)
CCB	Amlodipine	5mg	Oral/ Tablet	28	Zhejiang Jingxin	No	4.16
ACEI	Enalapril	5mg	Oral/ Tablet	16	Yangtze River	No	5.25
		10mg	Oral/ Tablet	16	Yangtze River	No	8.93
	Lisinopril	10mg	Oral/ Tablet	28	Zhejiang Huahai	No	6.45
	Fosinopril	10mg	Oral/ Tablet	14	Sino-American Shanghai Squibb	Yes	11.80
ARB	Losartan	50mg	Oral/ Tablet	7	Zhejiang Huahai	No	7.54
		50mg	Oral/ Tablet	14	Zhejiang Huahai	No	14.70
		100mg	Oral/ Tablet	7	Zhejiang Huahai	No	12.82
		100mg	Oral/ Tablet	14	Zhejiang Huahai	No	24.99
	Irbesartan	75mg	Oral/ Tablet	28	Zhejiang Huahai	No	5.66
		75mg	Oral/ Tablet	42	Zhejiang Huahai	No	8.37
ARB/diuretic combination	Irbesartan and hydrochlorothiazide	150mg/12.5mg	Oral/ Tablet	14	Zhejiang Huahai	No	15.26
		150mg/12.5mg	Oral/ Tablet	28	Zhejiang Huahai	No	29.76

INN, international non-proprietary name. CCB, calcium channel blocker. ACEI, angiotensin-converting enzyme inhibitor. ARB, angiotensin receptor blocker.

Table S2. Subgroup analyses by sex: Comparison of the costs during one-year follow-up period between the pre-policy and post-policy cohorts

	**Female subgroup**					**Male subgroup**					**Between subgroup comparison (Male vs. Female)**		
**Costs**	**Pre-policy cohort (N=6937)**	**Post-policy cohort (N=8839)**	**Differences (post-policy vs. pre-policy cohort)**			**Pre-policy cohort (N=7623)**	**Post-policy cohort (N=10019)**	**Differences (post-policy vs. pre-policy cohort)**					
	Mean (SD)	Mean (SD)	Coefficient	P value	95% CI	Mean (SD)	Mean (SD)	Coefficient	P value	95% CI	Coefficient	P value	95% CI
**Hypertension-related costs, CNY**	1679.2 (2127.7)	1438.5 (1833.6)	-0.172	<0.0001	(-0.209, -0.136)	1919.0 (2617.1)	1571.4 (2557.1)	-0.214	<0.0001	(-0.256, -0.173)	-0.043	0.132	(-0.099, 0.013)
Costs for medicines	1508.6 (1682.3)	1286.6 (1552.9)	-0.176	<0.0001	(-0.209, -0.142)	1719.3 (2150.1)	1381.4 (1741.0)	-0.236	<0.0001	(-0.270, -0.201)	-0.062	0.011	(-0.111, -0.014)
NVBP antihypertensive medicines	717.5 (638.1)	458.1 (479.7)	-0.448	<0.0001	(-0.478, -0.417)	822.8 (744.2)	500.4 (539.7)	-0.496	<0.0001	(-0.526, -0.466)	-0.049	0.024	(-0.092, -0.006)
Other medicines	791.0 (1399.2)	828.5 (1389.0)	0.025	0.293	(-0.022, 0.073)	896.5 (1840.8)	881.0 (1559.2)	-0.041	0.127	(-0.095, 0.012)	-0.070	0.057	(-0.142, 0.002)
Costs for non-medicines	170.6 (961.4)	151.9 (682.8)	-0.154	0.050	(-0.308, 0.000)	199.7 (1132.7)	189.98 (1556.4)	-0.056	0.550	(-0.241, 0.128)	0.109	0.390	(-0.140, 0.358)
Costs on the health plan	934.2 (1424.7)	789.3 (1222.4)	-0.191	<0.0001	(-0.232, -0.151)	1041.3 (1874.0)	838.0 (1763.0)	-0.238	<0.0001	(-0.292, -0.184)	-0.046	0.187	(-0.116, 0.023)

Patient out-of-pocket costs	744.9 (895.9)	649.2 (760.2)	-0.150	<0.0001	(-0.187, -0.113)	877.7 (937.9)	733.4 (981.0)	-0.189	<0.0001	(-0.224, -0.154)	-0.041	0.113	(-0.092, 0.010)
**Non-hypertension-related costs, CNY**	7802.0 (16533.2)	8089.8 (19179.8)	-0.032	0.315	(-0.096, 0.031)	8267.7 (18353.9)	7951.9 (18257.7)	-0.067	0.051	(-0.134, 0.000)	-0.029	0.540	(-0.121, 0.064)
**All-cause costs, CNY**	9481.3 (16872.3)	9528.4 (19419.2)	-0.059	0.024	(-0.111, -0.008)	10186.7 (18848.0)	9523.4 (18758.3)	-0.099	<0.0001	(-0.153, -0.045)	-0.037	0.333	(-0.112, 0.038)

The relative differences were estimated using general linear models (GLM) with logarithmic link function and gamma distribution, and all baseline covariates were adjusted in the models. For between subgroup comparison, interaction term between the cohort and subgroup were added to the regression models, with Wald tests used to assess difference in policy effects between these subgroups.

Table S3. Subgroup analyses by age: Comparison of the costs during one-year follow-up period between the pre-policy and post-policy cohorts

	**Non-elderly subgroup (< 60 years)**					**Elderly subgroup (≥60 years)**							
**Costs**	**Pre-policy cohort (N=8258)**	**Post-policy cohort (N=10164)**	**Differences (post-policy vs. pre-policy cohort)**			**Pre-policy cohort (N=6302)**	**Post-policy cohort (N=8694)**	**Differences (post-policy vs. pre-policy cohort)**			**Between subgroup comparison (Elderly vs. Non-elderly)**		
	Mean (SD)	Mean (SD)	Coefficient	P value	95% CI	Mean (SD)	Mean (SD)	Coefficient	P value	95% CI	Coefficient	P value	95% CI
**Hypertension-related costs, CNY**	1587.9 (2256.0)	1381.5 (2345.2)	-0.170	<0.0001	(-0.210, -0.129)	2089.0 (2547.4)	1658.3 (2119.4)	-0.232	<0.0001	(-0.268, -0.195)	-0.068	0.017	(-0.124, -0.012)

Costs for medicines	1423.2 (1682.5)	1214.1 (1669.7)	-0.191	<0.0001	(-0.224, -0.158)	1875.4 (2215.3)	1480.6 (1628.6)	-0.234	<0.0001	(-0.268, -0.200)	-0.053	0.032	(-0.102, -0.005)
NVBP antihypertensive medicines	712.1 (668.9)	467.5 (509.2)	-0.427	<0.0001	(-0.456, -0.397)	851.9 (726.0)	495.9 (516.7)	-0.533	<0.0001	(-0.564, -0.502)	-0.110	<0.0001	(-0.153, -0.067)
Other medicines	711.0 (1377.8)	746.6 (1497.5)	-0.001	0.958	(-0.051, 0.048)	1023.5 (1928.0)	984.7 (1453.5)	-0.032	0.211	(-0.083, 0.019)	-0.042	0.255	(-0.114, 0.030)
Costs for non-medicines	164.6 (1173.1)	167.3 (1298.9)	-0.027	0.774	(-0.209, 0.156)	213.5 (874.8)	177.6 (1137.6)	-0.209	0.002	(-0.339, -0.078)	-0.200	0.109	(-0.445, 0.045)
Costs on the health plan	840.9 (1514.2)	714.8 (1622.7)	-0.199	<0.0001	(-0.249, -0.149)	1186.0 (1848.5)	932.5 (1413.6)	-0.244	<0.0001	(-0.289, -0.200)	-0.055	0.117	(-0.125, 0.014)
Patient out-of-pocket costs	746.9 (916.6)	666.7 (920.8)	-0.139	<0.0001	(-0.176, -0.102)	902.9 (918.2)	725.8 (841.0)	-0.215	<0.0001	(-0.248, -0.181)	-0.079	0.002	(-0.130, -0.028)
**Non-hypertension-related costs, CNY**	5840.7 (13358.2)	6052.1 (14901.1)	-0.036	0.268	(-0.099, 0.027)	10935.4 (21446.4)	10313.2 (22108.5)	-0.073	0.034	(-0.141, -0.005)	-0.045	0.346	(-0.138, 0.048)
**All-cause costs, CNY**	7428.6 (13777.9)	7433.6 (15342.8)	-0.066	0.010	(-0.117, -0.016)	13024.4 (21837.0)	11971.6 (22418.5)	-0.102	<0.0001	(-0.158, -0.046)	-0.041	0.287	(-0.116, 0.034)

The relative differences were estimated using general linear models (GLM) with logarithmic link function and gamma distribution, and all baseline covariates were adjusted in the models. For between subgroup comparison, interaction term between the cohort and subgroup were added to the regression models, with Wald tests used to assess difference in policy effects between these subgroups.

Table S4. Subgroup analyses by levels of healthcare institutions: Comparison of the costs during one-year follow-up period between the pre-policy and post-policy cohorts

Costs	Pre-policy cohort	Post-policy cohort	Relative differences (post-policy vs. pre-policy cohort)		
	Mean (SD)	Mean (SD)	Coefficient	P value	95% CI
Primary					
Number of patients	9498	12875	-	-	-
**Hypertension-related costs, CNY**	1634.5 (1693.0)	1350.2 (1562.2)	-0.204	<0.0001	(-0.231, -0.177)
Costs for medicines	1494.9 (1408.8)	1238.4 (1214.2)	-0.199	<0.0001	(-0.223, -0.175)
NVBP antihypertensive medicines	758.7 (675.3)	492.6 (497.2)	-0.433	<0.0001	(-0.458, -0.408)
Other medicines	736.1 (1065.1)	745.8 (982.6)	-0.006	0.727	(-0.040, 0.028)
Costs for non- medicines	139.6 (662.3)	111.7 (873.9)	-0.281	0.045	(-0.402, -0.006)
Costs on the health plan	937.7 (1133.3)	760.6 (1022.3)	-0.229	<0.0001	(-0.259, -0.199)
Patient out-of-pocket costs	696.8 (692.7)	589.5 (640.4)	-0.173	<0.0001	(-0.200, -0.146)
**Non-hypertension-related costs, CNY**	7914.1 (17518.5)	7890.0 (16106.8)	-0.083	0.053	(-0.137, 0.029)
**All-cause costs, CNY**	9548.6 (17770.3)	9240.2 (16332.3)	-0.081	0.124	(-0.151, 0.011)
Secondary					
Number of patients	2044	2387	-	-	-
**Hypertension-related costs, CNY**	1746.8 (2244.3)	1441.2 (2197.7)	-0.209	<0.0001	(-0.294, -0.125)
Costs for medicines	1540.8 (1477.1)	1245.3 (1436.5)	-0.246	<0.0001	(-0.306, -0.186)
NVBP antihypertensive medicines	764.0 (703.6)	425.0 (490.9)	-0.583	<0.0001	(-0.646, -0.517)
Other medicines	776.7 (1121.5)	820.2 (1242.7)	-0.008	0.838	(-0.072, 0.089)
Costs for non- medicines	206.0 (1297.6)	195.9 (1532.0)	-0.004	0.981	(-0.378, 0.387)
Costs on the health plan	924.0 (1594.5)	752.2 (1374.4)	-0.233	<0.0001	(-0.337, -0.129)

Patient out-of-pocket costs	822.8 (800.4)	688.9 (933.8)	-0.184	<0.0001	(-0.257, -0.112)
**Non-hypertension-related costs, CNY**	7410.4 (15457.8)	7601.0 (18382.3)	-0.095	0.181	(-0.235, 0.044)
**All-cause costs, CNY**	9347.8 (18792.9)	8851.6 (15885.8)	-0.122	0.033	(-0.234, -0.010)
Tertiary					
Number of patients	3018	3596	-	-	-
**Hypertension-related costs, CNY**	2379.9 (3862.9)	2123.4 (3754.2)	-0.146	<0.0001	(-0.221, -0.072)
Costs for medicines	2062.2 (3203.8)	1750.8 (2743.5)	-0.199	<0.0001	(-0.264, -0.132)
NVBP antihypertensive medicines	822.2 (758.1)	474.7 (576.0)	-0.555	<0.0001	(-0.608, -0.503)
Other medicines	1239.9 (2907.8)	1276.1 (2611.2)	-0.000	0.992	(-0.096, 0.095)
Costs for non- medicines	317.7 (1680.0)	372.5 (1884.7)	0.159	0.132	(-0.047, 0.367)
Costs on the health plan	1200.8 (2780.7)	1052.2 (2696.7)	-0.168	0.002	(-0.274, -0.063)
Patient out-of-pocket costs	1179.1 (1402.9)	1071.1 (1372.2)	-0.123	<0.0001	(-0.182, -0.065)
**Non-hypertension-related costs, CNY**	8761.6 (16856.4)	9946.3 (27222.4)	0.095	0.077	(-0.010, 0.201)
**All-cause costs, CNY**	11141.5 (17811.0)	12069.8 (27873)	0.042	0.328	(-0.042, -0.127)

The relative differences were estimated using general linear models (GLM) with logarithmic link function and gamma distribution, and all baseline covariates were adjusted in the models.

Table S5. Subgroup analyses by types of index medicines: Comparison of the medical costs during one-year follow-up period between the pre-policy and post-policy cohorts

**Costs**	**Pre-policy cohort**	**Post-policy cohort**	**Relative differences (post-policy vs. pre-policy cohort)**		
	Mean (SD)	Mean (SD)	Coefficient	P value	95% CI
CCB subgroup					
Number of patients	3449	5688	-	-	-
**Hypertension-related costs, CNY**	1771.3 (2767.5)	1504.2 (2801.3)	-0.195	<0.0001	(-0.258, -0.131)
Costs for medicines	1588.5 (2320.6)	1318.5 (1945.6)	-0.207	<0.0001	(-0.260, -0.155)

NVBP antihypertensive medicines	661.8 (596.5)	419.4 (470.4)	-0.460	<0.0001	(-0.504, -0.416)
Other medicines	926.6 (2108.3)	899.0 (1830.4)	-0.058	0.136	(-0.135, 0.018)
Costs for non- medicines	182.8 (1008.0)	185.7 (1691.0)	-0.137	0.325	(-0.411, 0.136)
Costs on the health plan	984.3 (2022.1)	810.0 (2007.6)	-0.240	<0.0001	(-0.324, -0.155)
Patient out-of-pocket costs	787.0 (937.1)	694.2 (969.8)	-0.145	<0.0001	(-0.195, -0.095)
**Non-hypertension-related costs, CNY**	8332.8 (17328.9)	8299.7 (17966.9)	-0.056	0.194	(-0.140, 0.028)
**All-cause costs, CNY**	10104.1 (17933.7)	9804.0 (18535.8)	-0.081	0.024	(-0.151, -0.011)
ACEI subgroup					
Number of patients	737	908	-	-	-
**Hypertension-related costs, CNY**	1691.5 (2691.8)	1410.8 (2163.8)	-0.242	<0.0001	(-0.375, -0.108)
Costs for medicines	1447.6 (1389.2)	1251.9 (1856.8)	-0.218	<0.0001	(-0.324, -0.113)
NVBP antihypertensive medicines	545.5 (523.8)	326.4 (369.0)	-0.536	<0.0001	(-0.642, -0.430)
Other medicines	902.0 (1097.4)	925.4 (1751.4)	-0.066	0.313	(-0.195, 0.063)
Costs for non- medicines	243.9 (2052.5)	158.9 (708.3)	-0.301	0.200	(-0.761, 0.160)
Costs on the health plan	882.6 (1314.2)	755.9 (1493.5)	-0.238	<0.0001	(-0.370, -0.107)
Patient out-of-pocket costs	787.0 (937.1)	694.2 (969.8)	-0.145	<0.0001	(-0.195, -0.095)
**Non-hypertension-related costs, CNY**	8510.2 (22013.9)	8579.5 (22654.6)	-0.037	0.707	(-0.232, 0.158)
**All-cause costs, CNY**	10104.1 (17933.7)	9804.0 (18535.8)	-0.081	0.024	(-0.151, -0.011)
ARB					
Number of patients	3972	4960	-	-	-
**Hypertension-related costs, CNY**	1700.0 (2004.2)	1457.6 (2068.0)	-0.174	<0.0001	(-0.227, -0.121)
Costs for medicines	1504.5 (1487.2)	1274.2 (1515.5)	-0.196	<0.0001	(-0.239, -0.154)
NVBP antihypertensive medicines	696.3 (627.1)	374.5 (431.1)	-0.624	<0.0001	(-0.667, -0.581)

Other medicines	808.1 (1182.5)	899.7 (1360.2)	0.071	0.012	(0.016, 0.126)
Costs for non- medicines	195.4 (980.4)	183.4 (1179.9)	-0.027	0.820	(-0.258, 0.205)
Costs on the health plan	936.3 (1403.7)	793.8 (1299.6)	-0.190	<0.0001	(-0.251, -0.129)
Patient out-of-pocket costs	787.0 (937.1)	694.2 (969.8)	-0.145	<0.0001	(-0.195, -0.095)
**Non-hypertension-related costs, CNY**	7792.9 (15444.2)	7850.7 (16919.3)	-0.049	0.225	(-0.129, 0.030)
**All-cause costs, CNY**	10104.1 (17933.7)	9804.0 (18535.8)	-0.081	0.024	(-0.151, -0.011)
ARB/diuretic combination subgroup					
Number of patients	1963	2395	-	-	-
**Hypertension-related costs, CNY**	1763.0 (2000.5)	1511.7 (1667.9)	-0.143	<0.0001	(-0.205, -0.081)
Costs for medicines	1609.7 (1653.9)	1382.4 (1483.0)	-0.141	<0.0001	(-0.199, -0.083)
NVBP antihypertensive medicines	700.8 (570.7)	512.7 (456.9)	-0.308	<0.0001	(-0.358, -0.257)
Other medicines	908.9 (1397.1)	869.7 (1289.8)	-0.026	0.538	(-0.107, 0.056)
Costs for non- medicines	153.2 (742.6)	129.2 (515.5)	-0.197	0.103	(-0.434, 0.040)
Costs on the health plan	973.5 (1345.3)	816.0 (1012.5)	-0.160	<0.0001	(-0.227, -0.092)
Patient out-of-pocket costs	789.4 (813.2)	695.6 (845.2)	-0.124	<0.0001	(-0.190, -0.058)
**Non-hypertension-related costs, CNY**	8266.6 (13932.8)	8200.6 (17953.9)	0.022	0.714	(-0.095, 0.138)
**All-cause costs, CNY**	10029.7 (14245.7)	9712.4 (18240.5)	-0.014	0.768	(-0.109, 0.081)

The relative differences were estimated using general linear models (GLM) with logarithmic link function and gamma distribution, and all baseline covariates were adjusted in the models.

Table S6. Sensitivity analyses: Baseline characteristics of the patients in the pre-policy and post-policy cohorts, after propensity score matching

Baseline characteristics	Matched pre-policy cohort (N=14286)	Matched post-policy cohort (N=14286)	p value
Age—mean (SD), years	56.7 (14.0)	56.6 (13.9)	0.341
Age group—no.(%), years			0.255
≤19	0 (0)	0 (0)	

20-29	120 (0.8)	118 (0.8)	
30-39	1,816 (12.7)	1,834 (12.8)	
40-49	2,745 (19.2)	2,875 (20.1)	
50-59	3,334 (23.3)	3,309 (23.2)	
60-69	3,638 (25.5)	3,657 (25.6)	
70-79	1,788 (12.5)	1,720 (12.0)	
≥80	845 (5.9)	773 (5.4)	
Male—no. (%)	7,492 (52.4)	7,446 (52.1)	0.586
Job—no. (%)			0.408
Working	7,093 (49.7)	7,163 (50.1)	
Retired	7,193 (50.4)	7,123 (49.9)	
Institution—no. (%)			0.067
Government organs and public institutions	3,103 (21.7)	2,949 (20.6)	
Company	11,159 (78.1)	11,317 (79.2)	
Others	24 (0.2)	20 (0.1)	
Medical history—no. (%)			
CCI—mean (SD)	0.74 (1.2)	0.74 (1.1)	0.754
Myocardial infarction	7 (0.1)	8 (0.1)	0.796
Stroke	267 (1.9)	244 (1.7)	0.305
Heart failure	68 (0.5)	70 (0.5)	0.864
Atrial fibrillation	56 (0.4)	58 (0.4)	0.851
Dyslipidemia	2,899 (20.3)	2,898 (20.3)	0.988
Diabetes	2,302 (16.1)	2,315 (16.2)	0.834
Chronic kidney disease	658 (4.6)	662 (4.6)	0.910
Baseline healthcare utilization			
Admissions—no. (%)	1214 (8.5)	1228 (8.6)	0.793
Number of outpatient visits—mean (SD)	28.2 (16.9)	28.1 (18.0)	0.455

Continuous data are presented as the mean (SD) and categorical data are presented as n (%). P values were computed by student's t tests for mean comparisons and by Chi-square tests for categorical variables. Significance level was settled as two-sided α < 0.05.

Table S7. Sensitivity analyses: Comparison of the adherence to antihypertensive medicines during one-year follow-up period between the matched pre-policy and post-policy cohorts for the main analysis, after propensity score matching

**Adherence**	**Matched pre-policy cohort (N=14286)**	**Matched post-policy cohort (N=14286)**	**Differences (post-policy vs. pre-policy cohort)**		
	Mean (SD)	Mean (SD)	Coefficient	p value	95%CI

Proportion of days covered (PDC) by NVBP medicines	0.28 (0.22)	0.31 (0.22)	0.020	<0.0001	(0.015, 0.025)
Patients who discontinued NVBP medicines—no.(%)	10700 (74.9)	10915 (76.4)	1.023	0.458	(0.988, 1.034)
Patients who used non-NVBP medicines—no.(%)	9527 (66.7)	8291 (58.0)	-0.372	<0.0001	(-0.421, -0.324)
Proportion of days covered (PDC) by non-NVBP medicines	0.27 (0.23)	0.26 (0.22)	-0.003	0.409	(-0.009, 0.004)
Patients who discontinued non-NVBP medicines—no.(%)	6345 (66.6)	5588 (67.4)	1.030	0.422	(0.899, 1.200)

PDC was calculated as the number of days covered by the medicine prescriptions divided by the one-year follow-up days. Discontinuation was defined as having a gap of more than 90 days since the end date of the last prescription, regardless of whether the therapy was resumed thereafter.

The differences in PDC and probability of using non-NVBP antihypertensive medicines were estimated using linear regressions and logit regressions, respectively, with adjustments for all baseline covariates.

Proportion of days covered (PDC) by non-NVBP antihypertensive medicines and proportion of patients who discontinued non-NVBP medicines were only estimated for patients who used these medicines during the one-year follow-up in each cohort.

Table S8. Sensitivity analyses: Comparison of the costs during one-year follow-up period between the matched pre-policy and post-policy cohorts for the main analysis, after propensity score matching

Costs	Matched pre-policy cohort (N=14286)	Matched post-policy cohort (N=14286)	Relative differences (post-policy vs. pre-policy cohort)		
	Mean (SD)	Mean (SD)	Coefficient	p value	95%CI
**Hypertension-related costs, CNY**	1806.1 (2375.6)	1473.3 (2092.8)	-0.198	<0.0001	(-0.227, -0.169)
Costs for medicines	1621.8 (1926.9)	1309.7 (1594.9)	-0.209	<0.0001	(-0.235, -0.183)
NVBP medicines	776.1 (697.7)	478.9 (514.6)	-0.470	<0.0001	(-0.493, -0.448)
Non-NVBP medicines	845.6 (1627.1)	830.8 (1411.5)	-0.014	0.475	(-0.053, 0.025)
Costs for non- medicines	184.2 (1050.8)	163.5 (1003.3)	-0.125	0.044	(-0.246, -0.003)
Costs on the health plan	991.4 (1656.4)	796.5 (1450.0)	-0.219	<0.0001	(-0.255, -0.182)
Patient out-of-pocket costs	814.6 (914.5)	676.7 (802.1)	-0.176	<0.0001	(-0.202, -0.150)
**Non-hypertension-related costs, CNY**	7992.8 (17302.3)	7583.4 (18197.2)	-0.045	0.078	(-0.095, 0.005)
**All-cause costs, CNY**	9798.9 (17715.8)	9056.7 (18563.6)	-0.078	<0.0001	(-0.118, -0.037)

The relative differences were estimated using general linear models (GLM) with logarithmic link function and gamma distribution, and all baseline covariates were adjusted in the models.

Checklist S1. STROBE Statement—Checklist of items that should be included in reports of *cohort studies*

	Item No	Recommendation	Page No
**Title and abstract**	1	(*a*) Indicate the study's design with a commonly used term in the title or the abstract	1
		(*b*) Provide in the abstract an informative and balanced summary of what was done and what was found	1
**Introduction**			
Background/rationale	2	Explain the scientific background and rationale for the investigation being reported	2-4
Objectives	3	State specific objectives, including any prespecified hypotheses	4
**Methods**			
Study design	4	Present key elements of study design early in the paper	5
Setting	5	Describe the setting, locations, and relevant dates, including periods of recruitment, exposure, follow-up, and data collection	4-5
Participants	6	(*a*) Give the eligibility criteria, and the sources and methods of selection of participants. Describe methods of follow-up	5-6
		(*b*) For matched studies, give matching criteria and number of exposed and unexposed	8-9
Variables	7	Clearly define all outcomes, exposures, predictors, potential confounders, and effect modifiers. Give diagnostic criteria, if applicable	6-7
Data sources/ measurement	8*	For each variable of interest, give sources of data and details of methods of assessment (measurement). Describe comparability of assessment methods if there is more than one group	6-7
Bias	9	Describe any efforts to address potential sources of bias	7
Study size	10	Explain how the study size was arrived at	9
Quantitative variables	11	Explain how quantitative variables were handled in the analyses. If applicable, describe which groupings were chosen and why	6-7

Statistical methods	12	(*a*) Describe all statistical methods, including those used to control for confounding	7-8
		(*b*) Describe any methods used to examine subgroups and interactions	7-8
		(*c*) Explain how missing data were addressed	NA
		(*d*) If applicable, explain how loss to follow-up was addressed	6
		(*e*) Describe any sensitivity analyses	8
**Results**			
Participants	13*	(a) Report numbers of individuals at each stage of study—eg numbers potentially eligible, examined for eligibility, confirmed eligible, included in the study, completing follow-up, and analysed	9
		(b) Give reasons for non-participation at each stage	9
		(c) Consider use of a flow diagram	In electronic supplementary material
Descriptive data	14*	(a) Give characteristics of study participants (eg demographic, clinical, social) and information on exposures and potential confounders	9
		(b) Indicate number of participants with missing data for each variable of interest	NA
		(c) Summarise follow-up time (eg, average and total amount)	6, 10
Outcome data	15*	Report numbers of outcome events or summary measures over time	21

*Give information separately for exposed and unexposed groups.

Note: An Explanation and Elaboration article discusses each checklist item and gives methodological background and published examples of transparent reporting. The STROBE checklist is best used in conjunction with this article (freely available on the Web sites of PLoS Medicine at http://www.plosmedicine.org/, Annals of Internal Medicine at http://www.annals.org/, and Epidemiology at http://www.epidem.com/). Information on the STROBE Initiative is available at http://www.strobe-statement.org.
